# Epidemiologic and economic impact of pharmacies as vaccination locations during an influenza epidemic

**DOI:** 10.1016/j.vaccine.2018.09.040

**Published:** 2018-10-16

**Authors:** Sarah M. Bartsch, Michael S. Taitel, Jay V. DePasse, Sarah N. Cox, Renae L. Smith-Ray, Patrick Wedlock, Tanya G. Singh, Susan Carr, Sheryl S. Siegmund, Bruce Y. Lee

**Affiliations:** a Public Health Computational and Operations Research (PHICOR), Johns Hopkins Bloomberg School of Public Health, Baltimore, MD, United States; b Global Obesity Prevention Center, Johns Hopkins Bloomberg School of Public Health, Baltimore, MD, United States; c Walgreens Center for Health & Wellbeing Research, Walgreens Company, Deerfield, IL, United States; d Pittsburgh Super Computing Center (PSC), Carnegie Mellon University, Pittsburgh, PA, United States; e Johns Hopkins Healthcare Solutions, Johns Hopkins University, Baltimore, MD, United States

**Keywords:** Influenza, Epidemic, Pharmacies, Vaccination, Economic

## Abstract

**Introduction:**

During an influenza epidemic, where early vaccination is crucial, pharmacies may be a resource to increase vaccine distribution reach and capacity.

**Methods:**

We utilized an agent-based model of the US and a clinical and economics outcomes model to simulate the impact of different influenza epidemics and the impact of utilizing pharmacies in addition to traditional (hospitals, clinic/physician offices, and urgent care centers) locations for vaccination for the year 2017.

**Results:**

For an epidemic with a reproductive rate (R0) of 1.30, adding pharmacies with typical business hours averted 11.9 million symptomatic influenza cases, 23,577 to 94,307 deaths, $1.0 billion in direct (vaccine administration and healthcare) costs, $4.2–44.4 billion in productivity losses, and $5.2–45.3 billion in overall costs (varying with mortality rate). Increasing the epidemic severity (R0 of 1.63), averted 16.0 million symptomatic influenza cases, 35,407 to 141,625 deaths, $1.9 billion in direct costs, $6.0–65.5 billion in productivity losses, and $7.8–67.3 billion in overall costs (varying with mortality rate). Extending pharmacy hours averted up to 16.5 million symptomatic influenza cases, 145,278 deaths, $1.9 billion direct costs, $4.1 billion in productivity loss, and $69.5 billion in overall costs. Adding pharmacies resulted in a cost-benefit of $4.1 to $11.5 billion, varying epidemic severity, mortality rate, pharmacy hours, location vaccination rate, and delay in the availability of the vaccine.

**Conclusions:**

Administering vaccines through pharmacies in addition to traditional locations in the event of an epidemic can increase vaccination coverage, mitigating up to 23.7 million symptomatic influenza cases, providing cost-savings up to $2.8 billion to third-party payers and $99.8 billion to society. Pharmacies should be considered as points of dispensing epidemic vaccines in addition to traditional settings as soon as vaccines become available.

## 1. Introduction

As getting people vaccinated as early as possible in an epidemic is crucial to mitigating the impact of an influenza epidemic [1,2], pharmacies may represent an important resource to increase reach and capacity of vaccine distribution in the event of a novel epidemic. Over the last century, four influenza pandemics have caused significant morbidity and mortality globally [3,4]. During the 2009 H1N1 pandemic, the federal government allotted vaccines to states based on population size, with each state determining where to administer their vaccine supply [5,6]. The relative epidemiological and economic benefits of using a single vaccination channel (traditional locations, such as doctor offices and hospitals) versus multiple channels (traditional locations plus alternative locations, such as pharmacies) to administer influenza vaccine in the event of an epidemic caused by a novel virus have not been determined.

Pharmacies are increasingly identified as key partners in public health, contributing to expanded patient access and emergency preparedness [7–10]. As of 2009, pharmacists in every US state are trained to vaccinate in some capacity [11–13]. However, despite nearly 86% of the population living within five miles of a pharmacy, 28.2% of adults and 4.9% of children received their seasonal influenza vaccine from a pharmacy in 2017 [14,15]. Pharmacies offer unique advantages that many traditional locations do not; they have expanded evening and weekend hours, provide vaccinations without an appointment, and are located in close proximity to patients, increasing access and convenience of immunization delivery [16–22]. To estimate the benefits of utilizing pharmacy locations in addition to traditional locations (e.g., doctor offices and hospitals) for immunization in the event of an influenza epidemic caused by a novel virus, we used the Public Health Influenza Laboratory agent-based model and the FluEcon clinical and economic outcomes model to simulate the spread of influenza and the impact of vaccination under varying conditions.

## 2. Methods

### 2.1. Public Health Influenza Laboratory (PHIL)

This version of the Public Health Influenza Laboratory (PHIL) is a refinement of the PHICOR and PSC team’s influenza agent-based model (ABM) described in previous publications [2,23–30]. PHIL utilizes a synthetic US population, developed by RTI International [31], which includes geographically placed representations of each person, household, workplace, and school for the year 2017. PHIL includes geographically explicit representations for six types of vaccination locations across the 48 continuous states: 5720 hospitals, 51,560 clinic and physician offices, 4659 urgent care clinics, and 61,202 pharmacies (21,781 large retailers and 39,421 others).

Each virtual agent in PHIL represents a person with a set of characteristics (e.g., age, sex, race, socioeconomic status) and is assigned to a geographically explicit household and, depending on age, workplace or school. PHIL simulates the movement of each agent from place to place (i.e., from household to work, from school to community, etc.) and the interactions between agents each day at each location. These interactions potentiate the transmission of influenza (Appendix Table A1).

Each agent could be in one of four mutually exclusive influenza states: (1) susceptible (S, not infected with influenza and able to become infected), (2) exposed (E, infected with influenza, but not able to transmit to others), (3) infectious (I, infected and able to transmit to others), or (4) recovered/immune (R, not infected and unable to become infected). All agents start in the ‘S state’. An influenza epidemic was seeded by randomly infecting 1000 agents on day one. The model advances in discrete one-day time steps, where agents interact with one another based on their movement between households, communities, workplaces, and schools. With each point of contact, agents in the ‘I state’ have a probability of transmitting influenza to agents in the ‘S state’, with those agents subsequently moving to the ‘E state’. An agent remains in the ‘E state’ for the latent period duration, before moving to the ‘I state’, where they are able to transmit to others. In the ‘I state’, 33% are asymptomatic, and are half as infectious as a symptomatic agent [32,33]. We assume 50% of all symptomatic agents stay home from school or work (2.5 and 1.5–5 days, respectively; Appendix Table A2), thereby limiting contact with other agents. The likelihood of influenza transmission between agents varies based on where contact occurs (Appendix Table A1), the reproductive rate (R0) (i.e., average number of secondary cases generated by one infectious case), the duration of infectiousness, and agent infectivity (i.e., if agent is symptomatic or asymptomatic). If an agent is vaccinated, the agent will have a probability of moving to the ‘R state’ based on the vaccine efficacy. We assume protection is complete and immediate (i.e., individuals move to the ‘R state’ on the day of vaccination).

Each agent’s likelihood of seeking vaccination is based on the distance they are willing to travel from their household to be vaccinated. Each day, an agent will look for a vaccination location within this set distance (i.e., radius) that varies for rural and urban areas; if no vaccination locations are within this radius, that agent is not eligible for vaccination. If more than one location is within the radius, an agent will randomly pick one location to visit on that day and will be vaccinated if there are enough doses available at that location. Age and gender limits were applied to specialized physician offices: women’s (only women), pediatric (0–18 years old), and geriatric (60 years and older) physician offices and clinics. Following current state regulations, only those of a certain age could be vaccinated in pharmacy locations. The number of people vaccinated each day at a given location depends on the location- specific daily vaccination rate. This rate is determined from number of persons that a nurse or pharmacist could vaccinate per hour (for pharmacies this was normalized by the weekly number of prescriptions filled to account for size) and the number of hours that location is open (Appendix Table A2). We assume hospitals and clinic and physician offices are open five days per week, urgent care centers could vaccinate six days per week, and pharmacies are open seven days per week.

### 2.2. FluEcon

Using PHICOR’s previously published FluEcon model [2,34], we translate the number of vaccinated persons and influenza infections from PHIL into health outcomes and their corresponding costs from the third-party payer and societal perspectives. Each symptomatic case has probabilities of seeking ambulatory care, being hospitalized, or dying from influenza. Each of these is associated with costs and health effects. The third-party payer perspective includes all direct costs (i.e., vaccination, ambulatory care, hospitalization). Societal costs include direct and indirect (i.e., productivity losses due to absenteeism and mortality) costs. Hourly wage for all occupations [35] serves as a proxy for productivity losses. Productivity losses for mortality result in the net present value of missed lifetime earnings based on annual wage [35] and years of life lost based on his/her life expectancy [36]. Health effects are measured in quality-adjusted life years (QALYs) and calculates QALYs lost (i.e., accounting for reductions in health effects due to influenza and/or death). Each person accrues QALY values based on age-dependent healthy QALY value attenuated by the influenza-specific utility weight for their illness duration. Death results in the loss of the net present value of QALYs for the remainder his/her lifetime.

For each scenario, we calculate the incremental cost-effectiveness ratio (ICER) and cost-benefit, as follows:

$$\text{ICER}=\frac{{\text{Cost}}_{A}-{\text{Cost}}_{B}}{{\text{Health Effects}}_{B}-{\text{Health Effects}}_{A}}$$

Cost-Benefit = Benefit – Cost = Direct Cost and Productivity Losses of Averted Influenza Cases – Additional Cost of Vaccinating in Pharmacies where A and B are two different scenarios (described below). ICERs <$50,000/QALY saved are considered cost-effective [37].

### 2.3. Data inputs and sources

Appendix Table A2 shows the model input parameters, values, and sources. Geospatial coordinates for traditional locations came from national databases [38–41], while coordinates for all pharmacy locations were provided by a large retail pharmacy. Data regarding the traditional locations were cleaned to only include locations that may distribute vaccines during an epidemic and to remove duplicates. Thus, the cleaned hospital dataset included all general acute care, children’s, military, veteran’s and women’s hospitals, while the physicians’ offices dataset included all primary care physicians, family practices, internal medicine practices, pediatricians, community health centers, geriatric clinics, and women’s clinics. Location coordinates and other data for all pharmacies came from internal and proprietary databases. Walgreens maintains an internal database which includes data such as each location’s address, pharmacy hours, and number of prescriptions sold per days. The proprietary database is developed by Walgreens based on national databases. We used data reported from February 2017. Each census tract was designated as urban, an urban cluster, or rural following the United States Department of Agriculture Economic Research Service [42]. The distance agents are willing to travel to be vaccinated is based on the distance persons traveled for medical or dental care from the National Household Travel Survey [43]. Minimum age for vaccination at pharmacy locations vary by state (ranging from 7 to 18 years), to be conservative we assumed the same minimum age of across all pharmacies. All costs, clinical probabilities, and durations came from the scientific literature or nationally representative data sources (e.g., Healthcare Cost and Utilization Project [44], CMS Physician Fee Schedule [45]). When available, all costs and probabilities are age-specific. All costs are 2017 $US, converted using a 3% discount rate.

### 2.4. Scenarios and sensitivity analyses

Our baseline scenario distributes vaccines only through traditional locations, while various experimental scenarios distributed vaccines through pharmacies in addition to traditional locations. We simulated vaccination in pharmacies two ways: (1) pharmacies have typical pharmacy hours (8–24 h, allowing them to potentially vaccinate more persons per day than traditional locations); and (2) pharmacies have extended hours, with all pharmacies initially open for <12 h now open for 12 h (i.e., all pharmacies able to vaccinate at least 12 h per day). These scenarios assume that each person seeking vaccination will be vaccinated (i.e., there are enough doses available to cover the population).

Each experiment consisted of 30 realizations in PHIL and Monte Carlo simulations of 1000 trials in FluEcon, varying each parameter throughout their ranges. Results are reported as mean and 95% credibility interval (CrI). Sensitivity analyses varied the R0 (1.30–1.63), the time between epidemic start and vaccine availability (1–28 days), and the probability of mortality (seasonal estimates and four times these values to simulate more virulent circulating strains of influenza [46]). We also evaluated the impact of various locations (traditional only, pharmacies only, and all locations) being able to vaccinate persons faster (i.e., increasing daily vaccination rate such as by adding additional staff per location) and the number and types of pharmacies that increase their hours (all large retail pharmacies and 20% of all other pharmacies). Additional scenarios evaluated the impact of limiting the number of doses to 50% of the needed supply, with doses distributed to locations based on the volume of people they could vaccinate in a day, so that larger locations received more vaccines. Other sensitivity analyses used total compensation (where wage represented 68.2% of the total value [47]) for productivity losses and included age-specific future medical costs [48], where the NPV of future medical costs were subtracted for those who die and accounted for the fraction of individuals that incur that cost at each age. We varied the total cost of the vaccine and its distribution in the cost-benefit analysis.

We ran these sensitivity analyses to account for variations in scenarios, such as epidemic severity, with R0 values within the range of reported values for past pandemics and seasonal influenza [49] and different mortality rates. Varying the availability of vaccines account for different situations like the epidemic starts elsewhere and US has forewarning, or we have future production, vaccine stockpiles (i.e. it is already available), or various timing for virus identification and vaccine distribution. Scenarios limiting the supply can account for stockpile depletion.

## 3. Results

### 3.1. Distribution of vaccines in traditional locations only

**Fig. 1.**
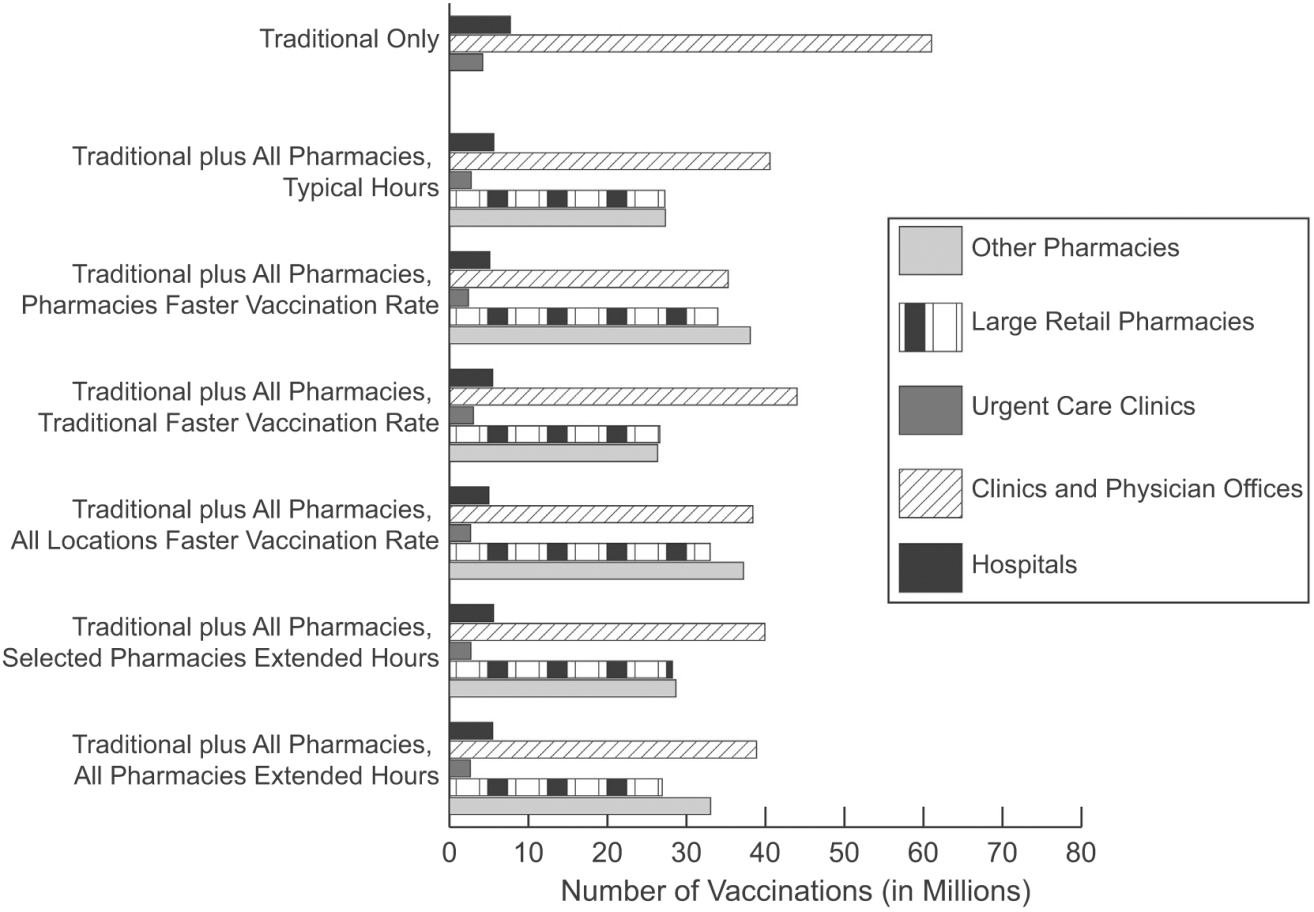
Total number of vaccinations administered by vaccination location type for a simulated novel influenza epidemic in the United States when vaccines are available 1 day after the epidemic start and there are enough doses for all persons. Traditional locations include 5720 hospitals, 51,560 clinic and physician offices, and 4659 urgent care clinics.

**Fig. 2.**
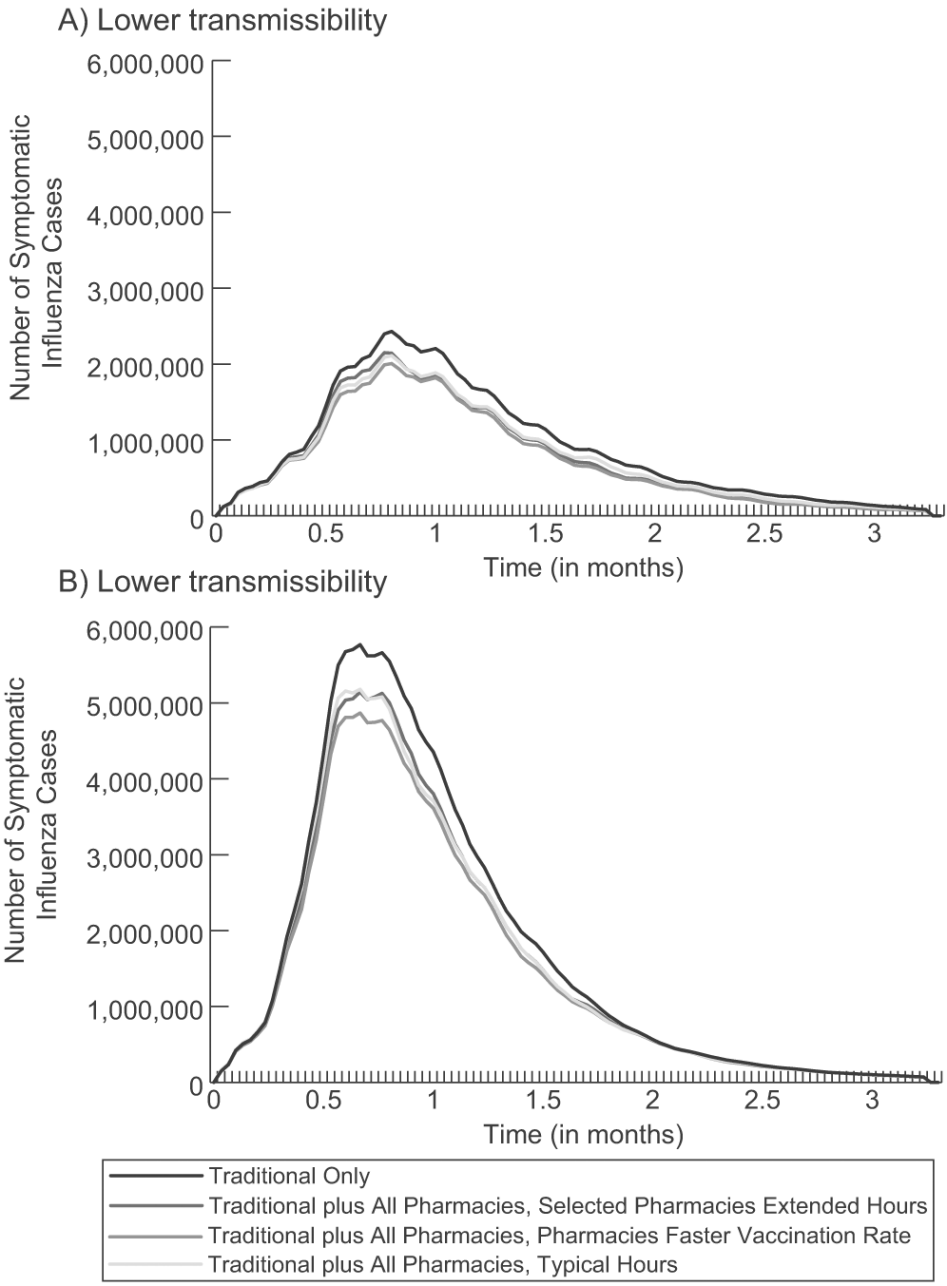
Number of new symptomatic influenza cases each day when simulating a novel influenza epidemic in the United States for a (A) an R0 of 1.30 and (B) an R0 of 1.63.

Traditional locations vaccinated 72,934,265 persons (23.8% coverage) when vaccines were available immediately after the epidemic start. This coverage results from most individuals only having access to one to two traditional locations that reach their maximum capacity of daily vaccinations (i.e., limited by location capacity not total number of available doses). Among the various traditional locations, clinic and physicians’ offices delivered the greatest number of vaccinations (Fig. 1). Direct vaccination costs totaled $3.4 billion (95% CrI: $3.2–3.5 billion). An epidemic with an R0 of 1.30 resulted in 87,008,275 symptomatic influenza cases nationwide with a total attack rate (i.e., symptomatic and asymptomatic infections) of 37.7%, while an R0 of 1.63 resulted in 167,084,786 symptomatic influenza cases with a total attack rate of 72.4% (Table 1); Fig. 2 shows the epidemic curve. Table 1 shows the number of deaths, QALYs lost, and economic outcomes.

Societal costs totaled $144.6–158.4 billion (seasonal to higher mortality, R0 of 1.30) to $296.8–315.4 billion (seasonal to higher mortality, R0 of 1.63) when considering compensation and future medical costs.

### 3.2. Distribution of vaccines in traditional locations and all pharmacy locations

**Fig. 3.**
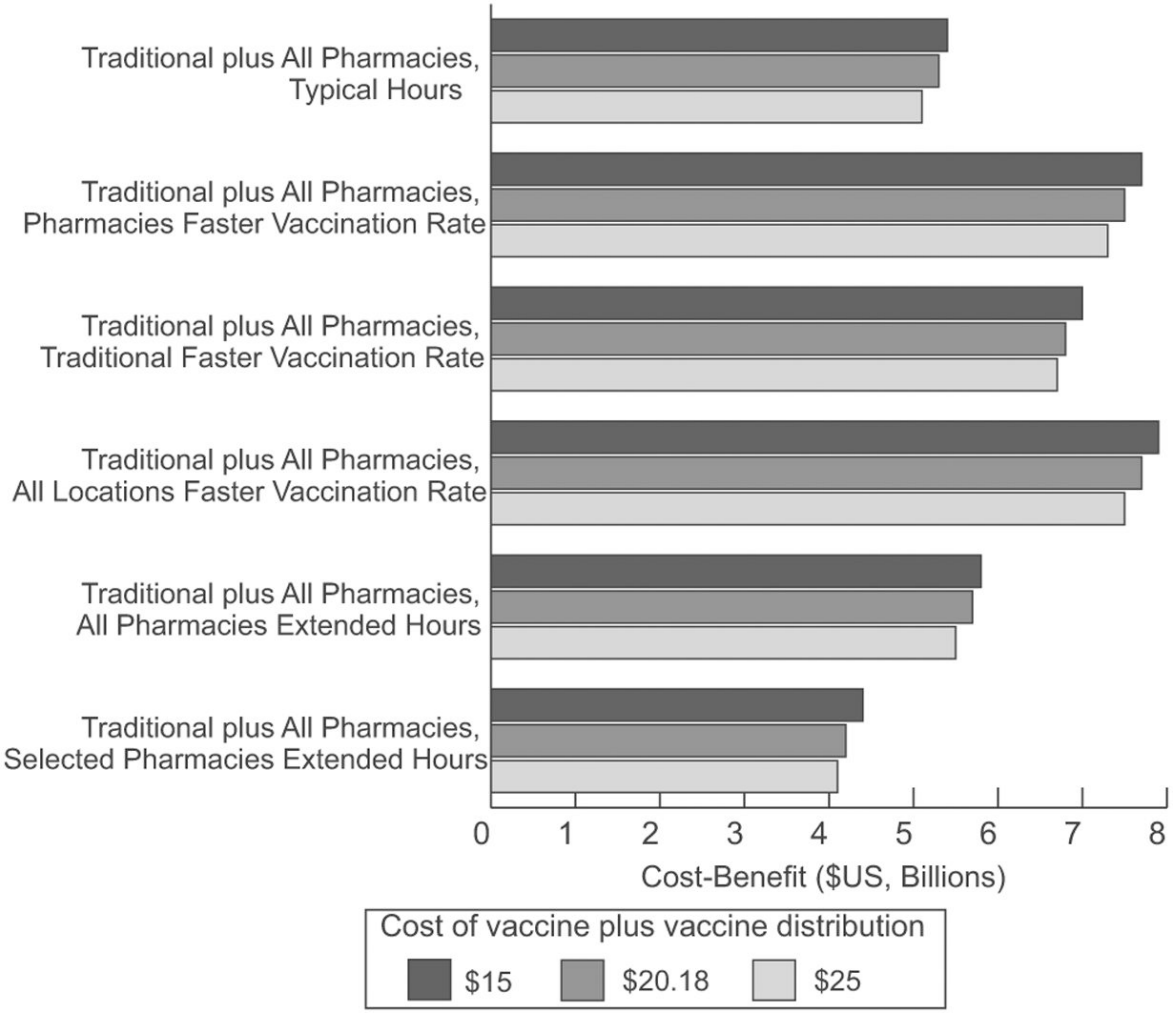
Cost-benefit ($US in billions) of distributing vaccines in traditional locations plus all pharmacy locations and in different ways compared to in traditional locations only for simulated novel influenza epidemic in the United States (R0 of 1.30, seasonal mortality rate) with various costing vaccines (a vaccine plus vaccine distribution cost of $20.18 represents base value; vaccine administration cost varied by vaccination location).

Table 1 shows the impact of distributing vaccines through all pharmacies under different conditions for both epidemic R0 values when vaccines were available the day after the epidemic start; Fig. 2 shows the epidemic curves. When all pharmacies were open their typical hours (i.e., 8–24 h), there were 103,530,217 total vaccinations (33.7% coverage) with 5,649,377 more vaccinations occurring in pharmacies than in traditional locations (Fig. 1). Vaccination in all locations led to 30.6 million more vaccinations than traditional locations alone, averting 11.9 million symptomatic influenza cases (Table 1) and 23,577 (95% CrI: 20,185–26,968) deaths. This resulted in cost-savings of $1.0 billion (95% CrI: $0.8–1.1 billion) from the third-party payer perspective and $5.2 billion (95% CrI: $3.8–6.6 billion) from the societal perspective, assuming a seasonal mortality rate. Increasing mortality (i.e., four times seasonal value) increased the amount of cost-savings, deaths, and QALY losses averted (Table 1). Adding all pharmacy locations was economically dominant (i.e., saved costs and provided health benefits) compared to vaccination in traditional locations only under all tested conditions. Fig. 3 shows the cost-benefit of distributing vaccines in pharmacy locations in addition to traditional locations for various vaccine and vaccine distribution costs. Adding all pharmacies (open their typical hours) resulted in a positive cost-benefit (i.e., net savings) totaling $7.5 billion (R0 = 1.30) to $7.6 billion (R0 = 1.63).

When delaying vaccine availability, vaccinating in all pharmacies open for their typical hours would avert 10.0 million influenza cases (available 14 days after epidemic start) and 6.2 million cases (available 28 days after epidemic start). Even with a delay in immunization, vaccinating in all pharmacies could avert 11,773 to 20,239 deaths, and save up to $25.0 million and $45.1 million from the third-party payer and societal perspectives, respectively and resulted in a cost-benefit of $4.3-$4.5 billion. Decreasing the number of doses (by at least 50%) did not have an impact on vaccination coverage, as there were still enough doses to keep up with the daily vaccination rate (i.e., rate of vaccination is the limiting factor).

Using compensation and accounting for future medical costs, vaccination in all locations resulted in cost-savings of $19.8–30.8 billion with no delay in availability and $13.3–18.1 billion with a delay, varying with R0 and mortality rate.

### 3.3. Impact of increasing rate at which people can be vaccinated at immunization locations

Vaccinating the maximum persons per hour in all pharmacies resulted in 114,833,820 vaccinations (37.4% coverage) and averted 17.1 million influenza cases (Table 1). When only traditional locations vaccinated the maximum per hour (assuming pharmacies had typical hours and baseline vaccination rate), there were 105,480,811 persons vaccinated (34.4% coverage; Fig. 1), averting 15.5 million influenza cases. When all locations vaccinate faster, there were 116,261,031 vaccinations (37.9% coverage), averting 17.8 million influenza cases, saving $1.4 billion and up to $7.6 billion from the third-party payer and societal perspectives, respectively. Distributing vaccines in traditional and pharmacy locations with a faster rate in any location was economically dominant compared to both distributing in only traditional locations and in all locations with the baseline vaccination rate from both perspectives for all epidemic conditions tested. Cost-benefit ranged from to $6.8–7.7 billion (Fig. 3).

Accounting for compensation and future medical costs, societal cost-savings totaled $25.1–28.4 billion (seasonal mortality) and $27.3–30.7 billion (higher mortality), regardless of which locations vaccinated at a faster rate, for an epidemic with an R0 of 1.30. For an epidemic with an R0 of 1.63, faster vaccination rates in all pharmacies saved $43.8–45.5 billion (varying with mortality rate).

### 3.4. Impact of extending pharmacy hours

Extending the hours of all large retailers and 20% of all other pharmacies (15,407 total with increased hours) resulted in 105.1 million vaccinations (Fig. 1) and averted 12.9 million symptomatic influenza cases compared to vaccination in only traditional locations. Cost-savings totaled $1.0 billion and $4.1–50.3 billion from the third-party payer and societal perspectives, respectively (Table 1). Extending the hours in all pharmacies resulted in 107.0 million vaccinations and averted 13.0 million cases, was economically dominant compared to traditional only and typical hours from both perspectives and resulted in cost-benefits (Fig. 3).

Extending hours in selected pharmacies garnered societal cost-savings of up to $20.1 billion (R0 = 1.30) and $31.8 billion (R0 = 1.63) when considering employee compensation and future medical costs.

## 4. Discussion

Our study shows that during an influenza pandemic, including pharmacies as vaccination locations could avert a substantial number of symptomatic influenza cases, deaths, and costs. Our results show the value of vaccination during a novel influenza epidemic depends on the number of vaccination locations, as the impact increases with additional sites (i.e., pharmacies). This is consistent with the observation that proportions of the population do not have ready or convenient access to health clinics and hospitals. Vaccine administration rate also plays an important role. Health clinics and hospitals may not have the capacity to vaccinate enough people early enough during the epidemic. Pharmacies have the potential advantage of being more focused on dispensing medications and vaccines during an epidemic without having the same range of services that a traditional location needs to provide [8]. Consequently, the rate at which people can be immunized and capacity may be proportionately higher in pharmacies. Finding ways to further increase pharmacy vaccination rates, such as training pharmacy technicians to vaccinate patients or creating special queues, could be important and may further increase the value of pharmacies.

Pharmacies have potential advantages as immunization sites, including numerous locations in closer proximity to residential neighborhoods, extended operating hours seven days a week, and ability to serve individuals on a walk-in basis, including those without an established healthcare provider [16,18,22,50]. Another advantage is improved access, especially to those residing in medically underserved areas [17,21,51]. Our results show that expanded access and convenience of pharmacy vaccination increases vaccination coverage (33.7% vs. 23.8% when including all pharmacies open typical hours). Additionally, pharmacies may be able to administer vaccinations at a reduced cost compared to traditional locations [52,53].

However, there are limitations to pharmacies. Although pharmacists are authorized to administer vaccinations, there may be policies which limit that authority. In the case of an epidemic, protocols may limit the ability for pharmacies to vaccinate. For example, state-imposed age restrictions limits pharmacists’ ability to vaccinate children. If age restrictions are lowered or lifted during an epidemic, pharmacy locations may further increase vaccination access, especially for those under the age of 18 in areas with few traditional locations. Additionally, vaccination in traditional locations can serve as a point entry for healthcare services that are not offered by pharmacies. Furthermore, some insurance providers do not reimburse for pharmacist-provided services.

There is ongoing national and state-level work to incorporate pharmacies into pandemic planning and the acceptability of pharmacists to serve as immunizers, with many pharmacies participating in a Centers for Disease Prevention and Control (CDC)-led effort along with government and private sectors [54]. Additionally, the current national pandemic plan lists continuing to work with pharmacies to improve operations for vaccine distraction as a key action [55]. Thus, quantifying the potential value of pharmacies as vaccination locations can help a number of decision makers determine how to best leverage pharmacies in the event of an epidemic. According to Fitzgerald et al., underestimating the value of pharmacies is one of the biggest gaps in pandemic vaccine program planning [14]. Information on the resulting cost-savings can help policy makers and other officials determine how much can be invested into distributing and allocating vaccines to pharmacies. This also gives third-party payers a better sense of how to structure reimbursements for vaccines administered in other such locations. This information shows traditional locations that adding alternative locations is one way to reduce their burden. Reducing the vaccination workload of traditional locations may allow them to have a greater focus on patient care. It informs people there are more vaccination options out there that may be more convenient. It also shows pharmacies the value of providing this service; they can use this information to make the case for receiving epidemic vaccines and make decisions on program planning.

Models are simplifications of real life and cannot account for every possible event or outcome. The course of an actual epidemic may not conform to our model data and assumptions. Our scenarios assumed a novel virus for which there would be no residual immunity. Existence of residual immunity, such as what occurred during the 2009 pandemic and any other factors that may reduce transmission could result in lower attack rates. Moreover, measured attack rates, such as those previously reported [56], may not always represent actual attack rates. Our data inputs were derived from sources of varying rigor and quality; thus, our results may change as better data become available. For example, we used prescription fill rate to estimate pharmacy size and subsequent vaccination rate. If this proxy is inaccurate, the value of pharmacies would fluctuate based on with the number of vaccines pharmacies could administer (i.e., fewer doses reduces value). Our model did not account for individuals’ potential vaccination location preference and assumes that vaccination likelihood was not dependent on type and number of locations in the area (besides the age/gender limits imposed on specialty practices). However, the presence of clinicians may be associated with the probability of receiving vaccines [57]. Thus, this preference may reduce the value of adding pharmacies if a person is only willing to be vaccinated by their own physician. Our scenarios made vaccines available at the same time, regardless of location. However, during the 2009 H1N1 pandemic, vaccines were not made available in pharmacies until much later than traditional locations [58]. A delay in vaccine availability for pharmacies would lower the incremental benefit of including pharmacies. Our model focuses only on traditional locations that have an established channel for distributing vaccinations that may vaccinate during an epidemic and does not include other potential locations (e.g., workplaces and schools), as these locations require special set up, such as a new distribution chain. As our study focuses on vaccination and determining if adding locations would be helpful, we did not evaluate the distribution of antivirals (which could also affect the spread of influenza) nor did we include mass social distancing measures, such as school closures. Given our study focus, a delay in vaccine availability for all locations would reduce the overall value of vaccination (i.e., epidemic not mitigated to the same degree) but would still show value of adding pharmacies.

Furthermore, we made several assumptions that may impact the value of vaccinations. For example, we assumed an equal chance of visiting any of the eligible vaccination location types (if all types are available to a person), as capturing preference for each person is complicated. We also assumed each location type will have an epidemic vaccine supply. However, not all locations may be willing to offer vaccines or be willing to offer them to non-patients. For example, not everyone could be vaccinated in all locations (we may not account for all limitations for each location) - hospitals may not offer the vaccine to the general public, only vaccinating its patients and employees; physicians’ offices may not offer the vaccine to those who are not current patients. This assumption is conservative as it overestimates the value of vaccination in general, overestimates the benefits of traditional locations, and underestimates the value of pharmacies.

## 5. Conclusions

Administering vaccines through pharmacies in addition to traditional locations in the event of an epidemic can increase vaccination coverage and mitigate up to 23.7 million symptomatic influenza cases, providing cost-savings up to $2.8 billion to third-party payers and $99.8 billion to society. Pharmacies should be considered as points of administering epidemic vaccines in addition to traditional settings as soon as vaccines become available.

## Figures and Tables

**Table 1:** Total number of symptomatic influenza cases and mean (95% credibility interval) clinical outcomes and costs for a simulated novel influenza epidemic in the United States when distributing vaccines in traditional locations plus all pharmacy locations and in different ways.

Scenario	Total Symptomatic Influenza Cases	Total Deaths	QALYs Lost (Millions)	Costs ($US, Billions)		
				Direct (Third Party Payer Perspective)	Productivity Losses	Societal Perspective
**Epidemic R0 = 1.30** **Seasonal Mortality**						
*Traditional Only*	87,008,275	151,664(78,555–244,190)	2.0(1.2–3.1)	17.2(13.9–21.3)	31.4(0.6–66.2)	48.6(17.5–83.2)
*Traditional plus All Pharmacies*						
Typical Hours	75,109,773	128,088(66,392–206,092)	1.7(1.0–2.7)	16.2(13.4–19.7)	27.2(0.5–57.2)	43.4(16.4–73.4)
Typical Hours, Faster Rate	69,914,790	117,957(61,161–189,745)	1.5(0.9–2.5)	15.7(13.2–19.0)	25.4(0.5–53.3)	41.1(15.9–69.3)
Select Pharmacies Extended Hours	74,144,441	125,474(70,310–200,828)	1.6(1.0–2.5)	16.2(13.1–19.6)	28.3(1.9–56.5)	44.5(17.9–72.8)
**Higher Mortality**						
*Traditional Only*	87,008,275	606,657(314,220–976,758)	6.5(3.3–11.2)	17.2(13.9–21.3)	290.9(144.5–509.6)	308.0(162.0–526.5)
*Traditional plus All Pharmacies*						
Typical Hours	75,109,773	512,350(265,569–824,368)	5.5(2.8–9.5)	16.2(13.4–19.7)	246.5(122.6–431.0)	262.7(139.1–447.2)
Typical Hours, Faster Rate	69,914,790	471,830(244,643–758,982)	5.1(2.6–8.7)	15.7(13.2–19)	227.4(113.3–397.1)	243.1(129.2–413.0)
Select Pharmacies Extended Hours	74,144,441	501,896(281,240–803,311)	5.4(2.9–9.1)	16.2(13.1–19.6)	241.6(119.4–410.5)	257.7(135.5–427.5)
**Epidemic R0 = 1.63** **Seasonal Mortality**						
*Traditional Only*	167,084,786	326,043(180,888–523,510)	4.1(2.4–6.4)	31.6(23.9–40.3)	62.1(4.4–123.9)	93.7(36.4–155.9)
*Traditional plus All Pharmacies*						
Typical Hours	151,070,995	290,636(161,372–466,580)	3.6(2.2–5.8)	29.7(22.9–37.5)	56.1(4–111.6)	85.9(34.1–141.8)
Typical Hours, Faster Rate	143,374,308	289,444(160,718–464,659)	3.6(2.2–5.7)	29.7(22.9–37.5)	55.9(3.9–111.2)	85.7(34.1–141.4)
Select Pharmacies Extended Hours	150,597,646	273,485(151,916–438,982)	3.4(2.1–5.4)	28.8 (22.3–36)	53.3(3.7–105.9)	82.1(32.9–135.3)
**Higher Mortality**						
*Traditional Only*	167,084,786	1,304,170(723,551–2,094,041)	13.6(7.2–23.5)	31.6(23.9–40.3)	611.6(298–1,049.8)	643.1(329.9–1,079.9)
*Traditional plus All Pharmacies*						
Typical Hours	151,070,995	1,162,545(645,487–1,866,320)	12.2(6.4–20.9)	29.7(22.9–37.5)	546.1(266.4–936.3)	575.8(296.1–965.2)
Typical Hours, Faster Rate	143,374,308	1,157,777(642,873–1,858,635)	12.1(6.4–20.8)	29.7(22.9–37.5)	543.9(265.4–932.5)	573.6(295–961.4)
Select Pharmacies Extended Hours	150,597,646	1,093,942(607,665–1,755,929)	11.5(6.1–19.7)	28.8(22.3–36)	514.5(251.2–881.4)	543.3(279.7–909.9)

Note: Costs in 2017 $US; Traditional locations include 5720 hospitals, 51,560 clinic and physician offices, and 4659 urgent care clinics; Faster rate = 23 persons per hour in all large retailers and 15 per hour in other pharmacies; Select pharmacies extended hours = all large retailers and 20% of all other pharmacies open for 12 or more hours (all others are open typical hours); Higher mortality is four times seasonal mortality estimates to simulate more virulent circulating strains of influenza.

## Appendix A

See Tables A1 and A2.

### Table A1 PHIL contact and transmission parameters.

**Table:**

Contact group	Infectious individual	Susceptible individual	Transmission probability
Household	Adult	Adult	0.4
Household	Adult	Child	0.3
Household	Child	Adult	0.3
Household	Child	Child	0.6
Elementary school	Student	Student	0.0435
Middle school	Student	Student	0.0375
High school	Student	Student	0.0315
Workplace	Adult	Adult	0.0575
Hospital	Health care worker	Health care worker	0.0575
Hospital	Health care worker	Patient	0.01
Hospital	Patient	Health care worker	0.01
Community	All	Adult	0.00480
Community	All	Child	0.00255
Social network	Location	Individual	Mean contacts per day
School	Classroom	Student	13.5
School	Outside classroom	Student	15
Community (weekday)	Outside of school	Student	16.2
Community (weekend)	Outside of school	Student	24.3
Community	Community	All	32.4
Workplace	Within office	Worker	2
Workplace	Outside of office	Worker	8
Health care facility	Within ward or clinic	Health care worker	2
Health care facility	Outside ward or clinic	Health care worker	8
Health care facility	With patients	Health care worker	30

### Table A2 Model input parameters, values, and sources.

**Table:**

Parameter	Mean or median	Standard error or range	Source
***Other ABM Inputs***			
Radius of distance travel for vaccination			
Urban	7.4		[43]
Urban cluster	11.7		[43]
Rural	16.1		[43]
Persons vaccinated per hour			
Hospitals	25		[51]
Physician offices, clinics, and urgent care centers		8–12	[51]
Large retail pharmacies		3–23	[51]
Other pharmacies		1–15	[51]
Hours open per day			
Hospitals	8		Assumption
Urgent care	12		Assumption
Clinics and physician offices	8		Assumption
Large retail pharmacies	12.6	8–24^†^	Pharmacy Database^◊^
Other pharmacies	8.62		Pharmacy Database^◊^
Prescriptions filled per day	182	17–1,496	Pharmacy Database^◊^
Vaccine efficacy	0.62^††^		[59]
***Costs (2017 US$)***			
Vaccine	18.36		[60]
Vaccine transport and storage (per vaccine)	1.82		[61]
Vaccine administration			
Traditional location		23.26–28.42	[45]
Pharmacy*		18.00–21.00	Expert Opinion
Hourly wage (all occupations)^^^	18.34	11.95–29.79	[35]
Outpatient, given influenza	108.74		[45]
Hospitalization, given influenza			
<1 years old	5,548.87	358.00	[44]
1–17 years old	7,821.74	457.00	[44]
18–44years old	12,678.91	673.00	[44]
45–64years old	12,885.44	354.00	[44]
65–84years old	8,868.57	154.00	[44]
85+ years old	8,356.08	149.00	[44]
Mean annual medical expenses			
0–5 years old	3,207.70		[48]
6–17 years old	2,300.54		[48]
18–44 years old	4,113.73		[48]
45–64 years old	7,582.55		[48]
65+ years old	11,395.80		[48]
***Probabilities***			
Percent wage represents of total employee compensation	68.2		[47]
Symptomatic influenza	66.9	58.3–74.5	[62]
Missing work	72.0		[63]
Missing school	69.0		[64]
Ambulatory care, given influenza			
0–4 years old	45.5	9.8	[65]
5–17 years old	31.8	6.1	[65]
18–64 years old	31.3	1.4	[65]
65+ years old	62.0	2.7	[65]
Hospitalization, given influenza			
0–4 years old	1.41	0.47	[65]
5–17 years old	0.06	0.02	[65]
18–49 years old	0.42	0.14	[65]
50–64 years old	1.93	0.64	[65]
65+ years old	4.21	1.40	[65]
Mortality, given influenza			
0–4 years old	0.004	0.001	[65]
5–17 years old	0.001	0.000	[65]
18–49 years old	0.009	0.003	[65]
50–64 years old	0.134	0.045	[65]
65+ years old	1.170	0.390	[65]
***Durations (days)***			
Vaccine recipient time (h)			
Traditional location	1.14	0.17–2.0	[52]
Pharmacy	0.20	0.10–0.62	[52]
Ambulatory care	0.5		Assumption
Work missed		1.5–4.9	[66]
School missed	2.54		[64]
Duration of symptoms	7.0		[67]
Hospitalization			
<1 years old	2.8	0.1	[44]
1–17 years old	3.1	0.1	[44]
18–44 years old	4.6	0.2	[44]
45–64 years old	5.2	0.1	[44]
65–84 years old	4.2	0.1	[44]
85+ years old	4.5	0.1	[44]
***Utility weights***			
Healthy QALY			
<17 years old	1.00		[68]
18–64 years old	0.92		[68]
65+ years old	0.84		[68]
Influenza without hospitalization	0.648	0.103	[69–78]
Influenza with hospitalization	0.514	0.089	[71,78,79]

† For Walgreens pharmacies this varies by locations; values are range across all locations.

†† Representative across various seasonal influenza vaccine presentations, ages, and years.

◊ Data for Walgreens pharmacies come from an internal database maintained by Walgreens, while data on other pharmacies come from a proprietary databased developed by Walgreens with sources such as the National Counsel for Prescription Drug Program. Data accessed February 2017.

* Includes supplies and pharmacist time.

^ Values are median and interquartile range (25% and 75%).
